# Supplementary material for: End Sequence Analysis Toolkit (ESAT) expands the extractable information from single-cell RNA-seq data
Source: Genome Res. 2016 Oct;26(10):1397–410. doi: 10.1101/gr.207902.116 (PMC5052061; doi:10.1101/gr.207902.116)
Supplement: Supplemental Material [file supp_gr.207902.116_Supp_Materials.tar.gz › ESAT-esat_v0.1/doc/html/classumms_1_1core_1_1annotation_1_1_single_interval-members.html]

ESAT: Member List


ESAT


- Main Page
- Related Pages
- Packages
- Classes
- Files

- Class List
- Class Index
- Class Hierarchy
- Class Members

All Classes Namespaces Files Functions Variables Enumerator Pages

- umms
- core
- annotation
- SingleInterval

umms.core.annotation.SingleInterval Member List

This is the complete list of members for umms.core.annotation.SingleInterval, including all inherited members.

|  |  |  |
| --- | --- | --- |
| compareTo(SingleInterval other) | umms.core.annotation.SingleInterval |  |
| contains(SingleInterval other) | umms.core.annotation.SingleInterval |  |
| equals(SingleInterval other) | umms.core.annotation.SingleInterval |  |
| getDistanceTo(SingleInterval other) | umms.core.annotation.SingleInterval |  |
| **getEnd**() (defined in **broad.core.datastructures.Interval.Impl**) | broad.core.datastructures.Interval.Impl |  |
| **getLength**() (defined in **broad.core.datastructures.Interval.Impl**) | broad.core.datastructures.Interval.Impl |  |
| **getRelationship**(Interval interval) (defined in **broad.core.datastructures.Interval.Impl**) | broad.core.datastructures.Interval.Impl |  |
| **getStart**() (defined in **broad.core.datastructures.Interval.Impl**) | broad.core.datastructures.Interval.Impl |  |
| HAS\_GREATER\_PART | broad.core.datastructures.Interval | static |
| HAS\_LESSER\_PART | broad.core.datastructures.Interval | static |
| HAS\_OVERLAPPING\_PART | broad.core.datastructures.Interval | static |
| **Impl**() (defined in **broad.core.datastructures.Interval.Impl**) | broad.core.datastructures.Interval.Impl |  |
| **Impl**(int start, int end) (defined in **broad.core.datastructures.Interval.Impl**) | broad.core.datastructures.Interval.Impl |  |
| intersect(SingleInterval other) | umms.core.annotation.SingleInterval |  |
| IS\_ADJACENT\_AND\_EMPTY | broad.core.datastructures.Interval | static |
| IS\_LEFT\_OVERHANGING\_OVERLAPPER | broad.core.datastructures.Interval | static |
| IS\_RIGHT\_OVERHANGING\_OVERLAPPER | broad.core.datastructures.Interval | static |
| IS\_STRICTLY\_GREATER | broad.core.datastructures.Interval | static |
| IS\_STRICTLY\_LESS | broad.core.datastructures.Interval | static |
| IS\_SUBSET | broad.core.datastructures.Interval | static |
| IS\_SUPERSET | broad.core.datastructures.Interval | static |
| **isAdjacent**(Interval interval) (defined in **broad.core.datastructures.Interval.Impl**) | broad.core.datastructures.Interval.Impl |  |
| overlaps(SingleInterval other) | umms.core.annotation.SingleInterval |  |
| SingleInterval() | umms.core.annotation.SingleInterval |  |
| SingleInterval(int start, int end) | umms.core.annotation.SingleInterval |  |
| SingleInterval(Interval other) | umms.core.annotation.SingleInterval |  |
| toString() | umms.core.annotation.SingleInterval |  |
| union(SingleInterval other) | umms.core.annotation.SingleInterval |  |


---

Generated on Fri Aug 15 2014 15:11:10 for ESAT by  

 1.8.7
